# Pairing in a dry Fermi sea

**DOI:** 10.1038/ncomms11875

**Published:** 2016-06-17

**Authors:** T. A Maier, P. Staar, V. Mishra, U. Chatterjee, J. C. Campuzano, D. J. Scalapino

**Affiliations:** 1 Computer Science and Mathematics Division, Center for Nanophase Materials Sciences, Oak Ridge National Laboratory, 1 Bethel Valley Road, PO Box 2008, Oak Ridge, Tennessee 37831-6494, USA; 2 IBM Research—Zürich, CH-8803 Rüschlikon, Switzerland; 3 Joint Institute of Computational Sciences, University of Tennessee, Knoxville, Tennessee 37996, USA; 4 Center for Nanophase Materials Sciences, Oak Ridge National Laboratory, Oak Ridge, Tennessee 37831, USA; 5 Department of Physics, University of Virginia, Charlottesville, Virginia 22904-4714, USA; 6 Department of Physics, University of Illinois at Chicago, Chicago, Illinois 60607, USA 22904; 7 Department of Physics, University of California, Santa Barbara, California 93106-9530, USA

## Abstract

In the traditional Bardeen–Cooper–Schrieffer theory of superconductivity, the amplitude for the propagation of a pair of electrons with momentum **k** and −**k** has a log singularity as the temperature decreases. This so-called Cooper instability arises from the presence of an electron Fermi sea. It means that an attractive interaction, no matter how weak, will eventually lead to a pairing instability. However, in the pseudogap regime of the cuprate superconductors, where parts of the Fermi surface are destroyed, this log singularity is suppressed, raising the question of how pairing occurs in the absence of a Fermi sea. Here we report Hubbard model numerical results and the analysis of angular-resolved photoemission experiments on a cuprate superconductor. In contrast to the traditional theory, we find that in the pseudogap regime the pairing instability arises from an increase in the strength of the spin–fluctuation pairing interaction as the temperature decreases rather than the Cooper log instability.

## Abstract (web summary)

Pairing interaction appears at room temperature in traditional superconductors with a Cooper instability in the Fermi sea. Here, Maier *et al.* report that in the pseudogap phase of cuprate, where this instability is absent, superconductivity arises from an increase in the strength of the spin fluctuation pairing interaction as the temperature decreases.

## Graphical abstract

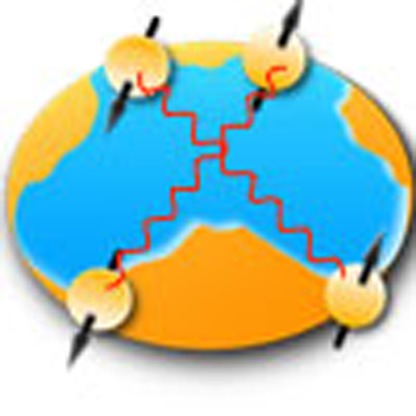

Evidence that the pseudogap (PG) in a slightly underdoped Bi_2_Sr_2_CaCu_2_O_8+*δ*_ (Bi2212, *T*_c_∼90 K) sample destroys the Bardeen–Cooper–Schrieffer (BCS) logarithmic pairing instability^1^ raises again the question of the role of the PG in the high-temperature superconducting cuprates^2^. The elimination of the BCS instability is consistent with the view that the PG competes with superconductivity. However, as noted in ref. 1, the onset of superconductivity with a *T*_c_∼90 K suggests an alternative scenario in which the PG reflects the formation of short-range pairing correlations.

The superconducting transition temperature can be determined from the Bethe–Salpeter gap equation


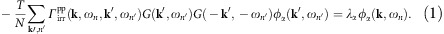


Here *G*(**k**, *ω*_*n*_) is the dressed single-particle Green's function, 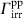 is the irreducible particle–particle pairing vertex, and **k** and *ω*_*n*_=(2*n*+1)*πT* are the usual momentum and Matsubara frequencies, respectively. The temperature at which the leading eigenvalue of equation (1) goes to 1 gives *T*_c_ and the corresponding eigenfunction *φ*_*α*_(**k**, *ω*_*n*_) determines the symmetry of the gap. In spin–fluctuation theories, the pairing vertex is approximated by an effective interaction


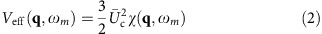


with *χ*(**q**, *ω*_*m*_) the spin susceptibility and 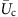 a coupling strength. Various groups have used experimental data to model *χ*(**q**, *ω*_*m*_), *G*(**k**, *ω*_*n*_) and 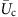, to determine whether a spin–fluctuation pairing interaction is consistent with the observed *T*_c_ values.

Dahm *et al.*^3^ used inelastic neutron scattering (INS) measurements for YBa_2_Cu_3_O_6.6_ to model the spin susceptibility *χ*(**q**, *ω*_*m*_) and a one-loop self-energy approximation to determine *G*. 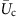 was an adjustable parameter estimated from INS and angular-resolved photoemission spectroscopy (ARPES) data. Using the resulting *G* and *V*_eff_ in equation (1), they concluded that a spin–fluctuation interaction had sufficient strength to account for the observed *T*_c_. Nishiyama *et al.*^4^ used INS results for *χ*(**q**, *ω*) and solved the Eliashberg equations for the heavy fermion compounds CeCuSi_2_ and CeIrIn_3_. For reasonable values of 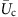, they found *T*_c_ values, which were again consistent with the notion that antiferromagnetic (AF) spin fluctuations were responsible for pairing in these materials. In a recent paper, Mishra, *et al.*^1^ used ARPES data at *T*=140 K for a slightly underdoped Bi2212 (*T*_c_=90 K) sample, to examine the effect of the PG on the superconducting transition temperature and to determine whether a spin–fluctuation pairing mechanism could account for the observed *T*_c_. They found that the usual BCS logarithmic divergence associated with the propagators in equation (1) was destroyed by the PG and the leading eigenvalue *λ*_d_(*T*) arising from the spin–fluctuation interaction (equation (2)) remained small, and was essentially independent of temperature. Thus, the question of the interplay between the PG and superconductivity^2^ continues to be of interest^5^,^6^,^7^,^8^.

Here we explore this question using numerical simulations for a Hubbard model and an analysis of ARPES data for a slightly underdoped Bi2212 sample in the superconducting state. The band parameters and filling of the Hubbard model are chosen so that it exhibits a PG as evidenced by a peak in the **q**=0 spin susceptibility, as the temperature decreases along with a gap, which opens in the antinodal region of the single-particle spectral weight *A*(**k**, *ω*). The ARPES data for Bi2212 was taken in the superconducting state at 40 K and provides a comparison with the previous analysis^1^ based on ARPES data taken in the normal state at 140 K. From the numerical calculations we conclude that the PG, similar to the superconductivity, arises due to short-range AF correlations, and that contrary to the usual case in which the pairing instability is associated with the Cooper instability, here the strength of the spin fluctuations increases as the temperature decreases, leading to the pairing instability. Our analysis of the 40 K Bi2212 ARPES data relative to the results of ref. 1, which were based on 140 K data on the same sample, shows an increase in the strength of the *d*-wave projection of the spin–fluctuation interaction strength, consistent with the dynamic cluster approximation (DCA) results. From this we conclude that contrary to the conclusion of ref. 1, spin fluctuations can account for the *d*-wave pairing in Bi2212.

## Results

### Underdoped Hubbard model with a PG

**Figure 1:**
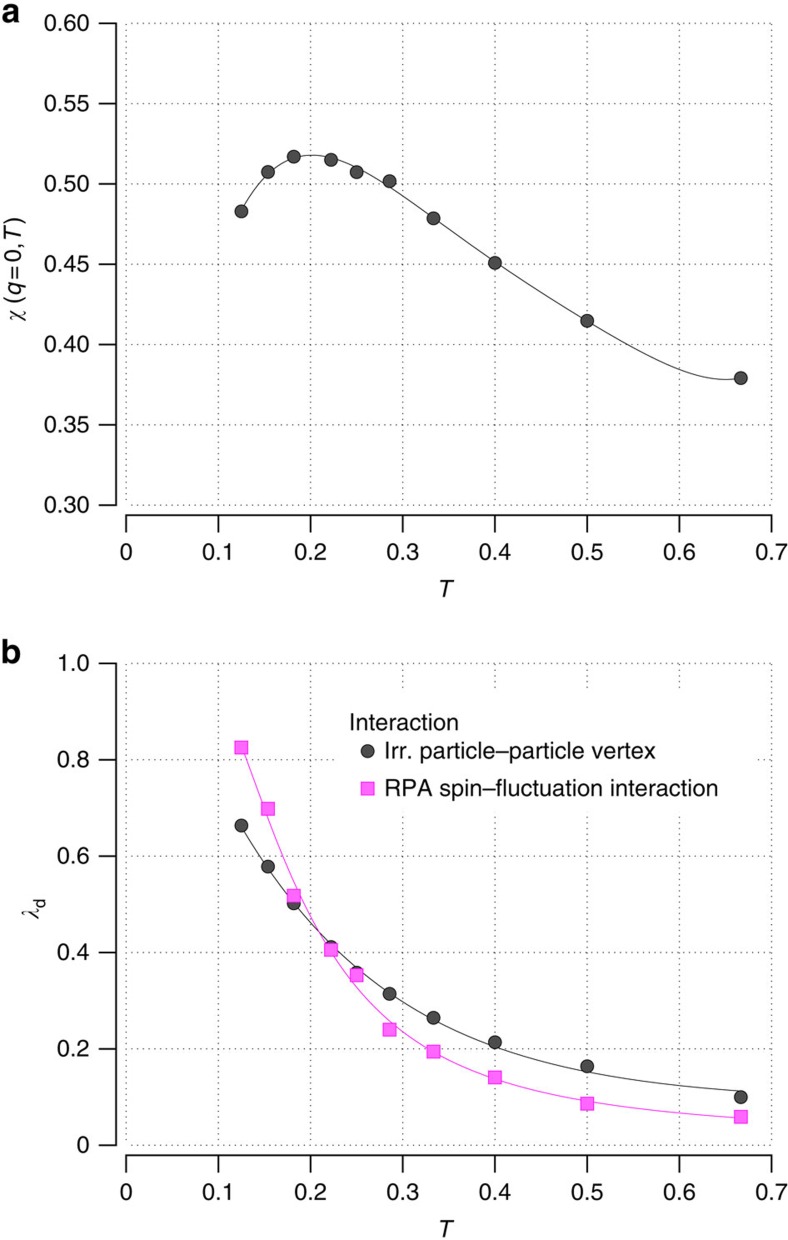
Pairing in the presence of a PG. (**a**) The uniform static spin susceptibility *χ*(**q**=0, *T*) versus temperature for 〈*n*〉=0.92 *t*′=−0.15 and *U*=7 peaks at a temperature T*=0.22 and decreases below this as the PG opens. (**b**) The leading eigenvalue *λ*_d_(*T*) of the particle–particle Bethe–Salpeter equation versus temperature (circles) from a DCA calculation of the irreducible particle–particle vertex 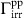. The *d*-wave eigenvalue for the spin–fluctuation interaction (equation (2)) with *χ*(**q**, *ω*_*m*_) the RPA spin susceptibility from equation (8) and 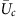
=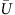
=6.7 is shown as solid squares. The solid lines are guides to the eye.

**Figure 2:**
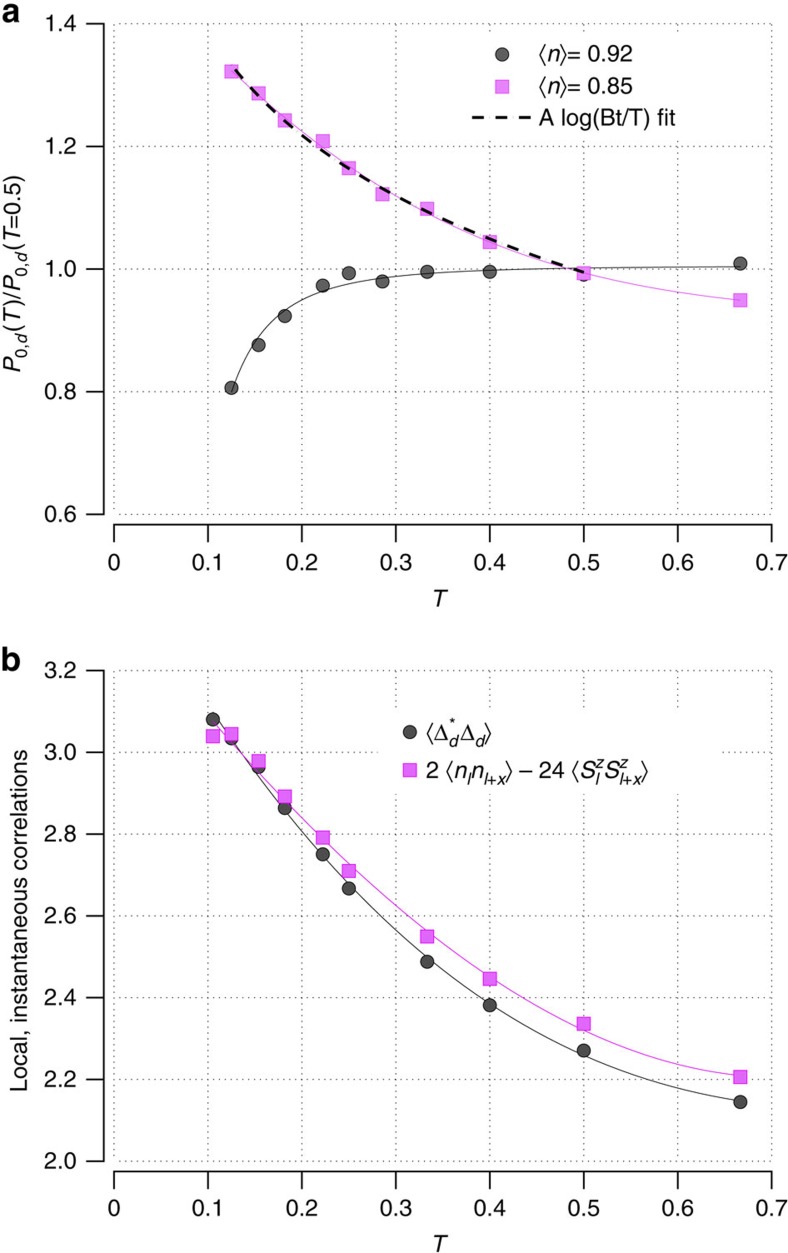
Destruction of the BCS logarithmic instability and nature of local pairing correlations. (**a**) The logarithmic BCS increase of the *d*-wave projection of the pairing kernel *P*_0,d_(*T*) for 〈*n*〉=0.92 is suppressed by the opening of the PG (circles). Here, *P*_0,d_(*T*) has been normalized to 1 at a temperature *T*=0.5*t* above the PG temperature T*. At temperatures below T*, where the PG has opened, the BCS logarithmic divergence is suppressed. The solid squares show *P*_0,d_(*T*) for a filling 〈*n*〉=0.85 where there is no PG and one sees the usual logarithmic increase as the temperature decreases. (**b**) The temperature dependence of the local *d*-wave pairfield correlation function 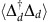 (circles). The observed increase in 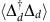 as *T* decreases below T* reflects the development of near-neighbour AF correlations (squares). The solid lines are guides to the eye.

**Figure 3:**
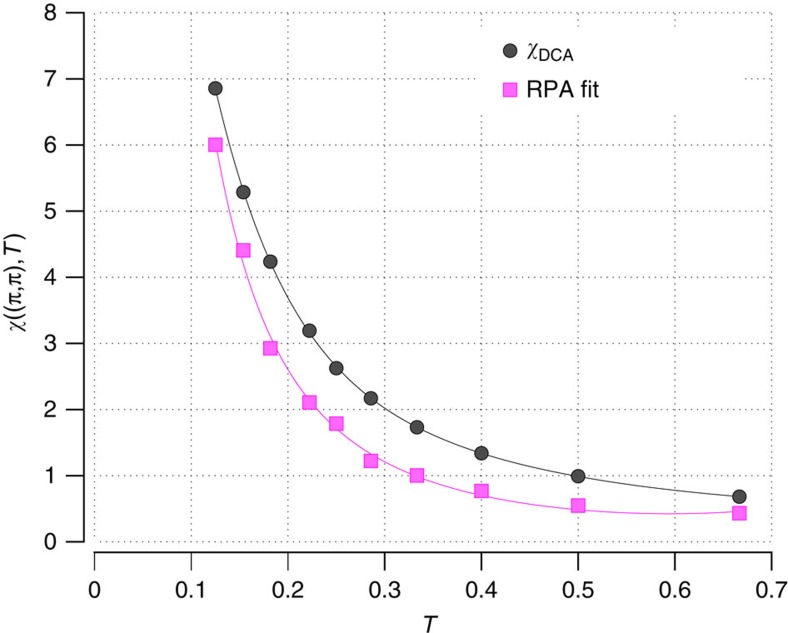
DCA spin susceptibility and RPA fit. The AF spin susceptibility *χ*_DCA_(**Q**=(*π*, *π*), *ω*_*m*_=0) from the DCA calculation (circles) and the RPA fit (equation (8)) with 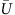
=6.7 (squares). The AF response continues to increase as *T* decreases below T*, leading to an increase of the spin–fluctuation interaction so that even though the BCS logarithmic increase of *P*_0_(*T*) is suppressed, the *d*-wave eigenvalue *λ*_d_(*T*) increases as seen in Fig. 1b. The solid lines are guides to the eye.

The two-dimensional Hubbard model we will consider has a near-neighbour hopping *t*, a next near-neighbour hopping *t*′/*t*=−0.15, an on-site Coulomb interaction *U*/*t*=7 and a filling 〈*n*〉=0.92. We will work in energy units where *t*=1. The DCA calculations^9^ were carried out on a 4 × 4 cluster and employed both continuous-time, auxiliary-field (CT-AUX) quantum Monte Carlo (QMC)^10^ and Hirsch-Fye (HF) QMC^11^ methods to solve the effective cluster problem. (The data in Figs 1, 2a and 3 were obtained with CT-AUX QMC and cross-checked with HF QMC. The equal-time data in Fig. 2b were obtained with HF QMC). (For more details, see Methods). In the DCA approximation, where 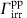 depends only on a finite set of cluster momenta **K**, the **k**-sum in equation (1) gives^12^


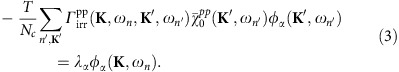


Here *N*_*c*_=16 is the cluster size and the pairing kernel *G*(**k**, *ω*_*n*_)*G*(−**k**, −*ω*_*n*_) has been coarse grained (averaged) over the momenta **k**′ of the DCA patches.


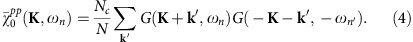


For the parameters we have chosen, the uniform static susceptibility *χ*(**q**=0, *T*) versus temperature, shown in Fig. 1a, exhibits a peak at T*=0.22 below which it decreases, as *T* is reduced^13^. This behaviour, seen in measurements of the magnetic susceptibility^14^ and Knight shifts^15^ of underdoped (hole) cuprates, reflects the opening of a PG. ARPES experiments^16^,^17^ find that this gap is anisotropic, opening in the antinodal regions of the Fermi surface. This behaviour has also been seen in DCA calculations of the single-particle spectral weight^13^,^18^. In Fig. 1b, the temperature dependence of the leading eigenvalue of the Bethe–Salpeter equation (3) is shown as the circles. Its eigenfunction has *d*-wave symmetry and *λ*_d_(*T*) approaches 1 at low temperatures. Thus, this model system has a PG that opens below T* and a *d*-wave eigenvalue that increases towards 1 as *T* decreases.

In addition to suppressing the **q**=0 spin susceptibility, we find that the opening of the PG destroys the low-temperature BCS logarithmic divergence of the *d*-wave projection of the pairing kernel


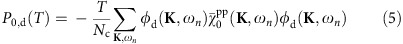


Here, 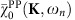 is defined in equation (4) and *φ*_d_(**K**, *ω*_*n*_) is the *d*-wave eigenfunction, which is approximated as


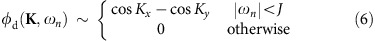


with *J*∼4*t*^2^/*U*. A plot of *P*_0,d_(*T*) versus *T* is shown in Fig. 2a and one can see that below T*, *P*_0,d_(*T*) is suppressed as the PG opens^19^,^20^. Here we have normalized *P*_0,d_(*T*) to its value at a temperature *T*=0.5*t* above T*. For comparison, the solid squares in Fig. 2a show *P*_0,d_(*T*) for 〈*n*〉=0.85, which does not have a PG and one sees the usual BCS logarithmic behaviour (dashed curve).

The absence of the BCS divergence in *P*_0,d_(*T*) when there is a PG is consistent with the finding of Mishra *et al.*^1^. *P*_0,d_(*T*) is the *d*-wave projection of what Mishra *et al.*^1^ referred to as the pairing kernel.) However, as noted, they found that with this suppression, the spin–fluctuation pairing interaction failed to give a superconducting transition. Based on this, they suggested that the PG reflects the presence of short-range pairfield correlations, which grow below T* and become coherent at *T*_c_. This behaviour could be likened to the magnetic response of the large *U* half-filled Hubbard model. In this case, the formation of local moments when the temperature drops below ∼*U*/2 is seen in an increase in the expectation value of the square of the local moment 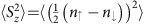. In a similar way, one can look for the onset of local pair formation as *T* decreases below the PG temperature T*. Here, with 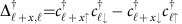 and 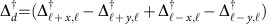, we have calculated 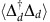 versus temperature. As shown in Fig. 1b, this correlation function does increase as the temperature decreases. However, the four near-neighbour pairfield correlations


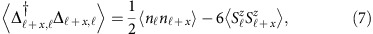


contribute the dominant contribution to this increase as shown in Fig. 2b. These results suggest that the PG is more closely related to the formation of short-range AF correlations than to local pair correlations in agreement with earlier ideas of Johnston^14^ and more recent theoretical results^5^,^6^,^7^,^8^,^21^. This identification of the PG with the development of short-range AF spin correlations is also consistent with the increase of the spin susceptibility *χ*(**q**=(*π*, *π*), *ω*_*m*_=0) as shown in Fig. 3 and as seen experimentally^22^.

### The spin–fluctuation pairing interaction

Returning to the question of whether the spin–fluctuation interaction (equation (2)) can lead to superconductivity when the logarithmic singularity of the BCS kernel is suppressed, we use DCA results for *G*(**k**, *ω*_*n*_) to construct *V*_eff_(**q**, *ω*_*m*_). Here, following Mishra *et al.*^1^, a random-phase approximation (RPA) form for *χ* is used


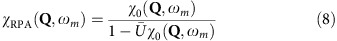


with


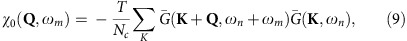


where 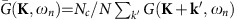 is the DCA coarse-grained Green's function (see Methods). The interaction 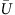 in the denominator of equation (8) is estimated from the approximate fit of *χ*_RPA_ to *χ*_DCA_ shown in Fig. 3.

Next, replacing 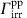 by *V*_eff_ in equation (2) with the coupling 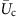 set to 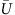 and using DCA Green's functions, we solve the Bethe–Salpeter equation (3). Results for *λ*_d_(*T*) are shown (solid squares) in Fig. 1b. We conclude that the increase in the strength of the pairing interaction *V*_eff_ leads to an increasing *λ*_d_(*T*) similar to that which is found using 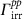 determined from the DCA calculation. Thus, in spite of the absence of the BCS logarithmic increase in *P*_0,d_(*T*), we find that the increase in the strength of the spin fluctuations leads to an increase in *λ*_d_(*T*) as the temperature is lowered.

This differs from the results of ref. 1, where ARPES data at *T*_0_=140 K was used to construct a single-particle *A*(**k**, *ω*, *T*_0_). Next, this spectral weight was used to approximate the single-particle propagator at lower temperatures *T*. In the Matsubara framework, this approximation is given by


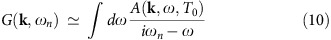


with *T*_0_=140 K and *ω*_*n*_=(2*n*+1)*πT*. Using this approximation, they found that the pairing kernel *P*_0,d_(*T*) (equation (7)) was essentially independent of temperature as opposed to decreasing with temperature as seen in the DCA calculation (Fig. 2a). We believe that this reflects a failure of the approximation (equation (10)), and that to determine *G*(**k**, *ω*_*n*_) at a temperature *T* one needs the spectral weight at *T*_0_=*T*.

To further explore this question, we have used Bi2212 ARPES data taken at 40 K to construct *G*(**k**, *ω*_*n*_) at *T*=40 K. This data was used earlier to study the neutron resonance in the superconducting state in ref. 23. We have used the same procedure to extract the single-particle spectral weight *A*(**k**, *ω*) from the raw data as in ref. 23. However, as detailed in the following, the contribution of the Gorkov Green's function *F*(**k**, *ω*) is estimated differently. Following ref. 1 we use an RPA form (equation (8)) for *χ*(**q**, *ω*_*m*_). At *T*=40 K, the system is superconducting so that


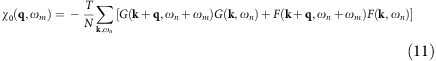


with *F*(**k**, *ω*_*n*_) the Gorkov Green's function.


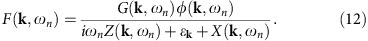


Here, *Z*(**k**, *ω*_*n*_) and *X*(**k**, *ω*_*n*_) are the even and odd parts of the ‘normal' self-energy and *φ*(**k**, *ω*_*n*_) is the gap function. For the band dispersion *ɛ*_**k**_, we used the ‘tb2' tight-binding fit of ARPES data on Bi2212 given in ref. 24. The frequency dependence of *X*(**k**, *ω*_*n*_) was neglected and *X*(**k**, 0) was lumped into a shift of the chemical potential and the tight-binding band structure parameters. Here we will make the additional approximation


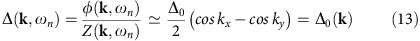


with *ɛ*_**k**_/*Z*(**k**, *ω*_*n*_)=*ɛ*_**k**_/*Z*_0_ and take *Z*_0_≈2 similar to ref. 1. In this case


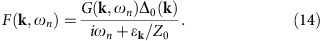


As in refs 1, 23, the phenomenological parameter 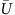 entering the spin susceptibility in equation (8) is determined by going to real frequencies and requiring that the **Q**=(*π*, *π*) spin resonance occurs at Ω∼40 meV. In our case, we find *U*≃500 meV.

**Figure 4:**
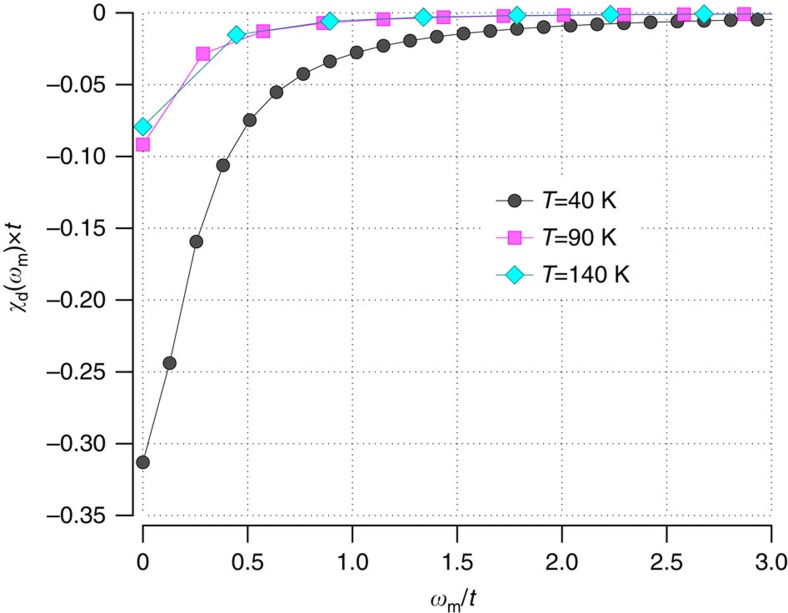
Temperature dependence of the spin–fluctuation interaction. *d*-wave projected spin susceptibility 〈*χ*(**k**−**k**′,*ω*_*m*_)〉_*d*_ (equation (15)) versus Matsubara frequency *ω*_*m*_ for *T*=40 K (black circles) calculated from the 40 K ARPES spectral weight *A*(**k**, *ω*, *T*=40 K), and for *T*=90 K (magenta squares) and 140 K (turquoise diamonds) calculated from *A*(**k**, *ω*, *T*=140 K). Consistent with the DCA results, the strength of the spin–fluctuation interaction increases as the temperature is lowered. The solid lines connect between the discrete Matsubara frequencies.

With these approximations, we have calculated *χ*(**q**, *ω*_*m*_) at *T*=40 K. The *d*-wave projection of *χ*(**q**, *ω*_*m*_)


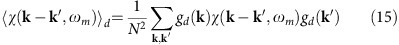


with *g*_*d*_(**k**)=cos *k*_*x*_−cos *k*_*y*_ is plotted in Fig. 4. This figure also shows results obtained at *T*=140 K, as well as at 90 K, using *A*(**k**, *ω*, *T*=140 K). Similar to the results for the temperature dependence of the pairing kernel, there is little change in 〈*χ*(**k**−**k**′, *ω*_*m*_)〉_*d*_ between 140 and 90 K based on *A*(**k**, *ω*, *T*=140 K). However, the *T*=40 K *d*-wave projection shows a clear increase in the strength of the spin fluctuations. We believe that if *A*(**k**, *ω*, *T*) spectral data were available for temperatures between 140 K and the superconducting transition temperature *T*_c_∼90 K, one would see the strength of the *d*-wave projection 〈*χ*(**k**−**k**′, *ω*_*m*_)〉_*d*_ increase, leading to an increase in the *d*-wave eigenvalue contrary to the results reported in ref. 1. One can of course ask whether the strength of the spin–fluctuation interaction at 40 K is sufficient to given an anti-nodal gap of order 50 meV. Using a value of the coupling constant 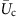 of order of the bandwidth 1.5 eV, the solution of the Gorkov equation


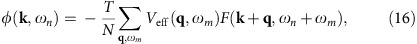


using the spin–fluctuation interaction *V*_eff_(*q*, *ω*_*m*_) in equation (2) and the approximation equation (14) for *F*, is found to give an anti-nodal gap of 50 meV.

## Discussion

We have used DCA calculations for an under (hole) doped 2D Hubbard model, which exhibits a PG, to see whether a spin–fluctuation interaction provides a reasonable approximation of the irreducible pairing interaction. In these calculations, the dynamic mean-field cluster is such that charge density and striping instabilities are suppressed, leaving AF and *d*-wave pairing as the dominant correlations. Although the PG eliminates the usual BCS logarithmic divergence of the pairing kernel, we find that a pairing instability arises from an increase in the strength of the spin–fluctuation interaction as the temperature decreases. The finding that the PG suppresses the BCS logarithmic divergence is similar to the result reported in ref. 1. However, the increase in the pairing strength of the spin fluctuations found in the DCA calculation is at odds with the results reported in ref. 1. We believe that this disagreement reflects a failure of the approximation in which the single-particle spectral weight at a higher temperature is used to determine the single-particle Green's function at lower temperatures.

Using a single-particle spectral weight constructed from ARPES data at 40 K, we find a significant enhancement of the *d*-wave projected spin–fluctuation strength relative to that at 140 K. Thus, we find that the DCA results are consistent with the 40 K ARPES data and the increase in the strength of the spin fluctuations can lead to superconductivity.

## Methods

### Hubbard model

The 2D Hubbard model we consider in the numerical calculations is described by the Hamiltonian


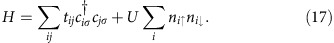


Here, 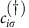 destroys (creates) an electron with spin *σ* on site *i* and 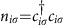 is the corresponding number operator. The hopping *t*_*ij*_ has a near-neighbour amplitude *t* and a next-near-neighbour amplitude *t*′=−0.15*t*, leading to a dispersion


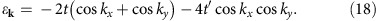


We use *t*=1 as the unit of energy and set the Coulomb repulsion *U*=7*t*.

### Dynamic cluster approximation

To study the behaviour of the Hubbard model in equation (17), we use a DCA QMC algorithm^9^. Similar to finite-size lattice calculations, the DCA represents the bulk lattice by a finite-size cluster, but uses coarse graining to retain information about the bulk lattice degrees of freedom not represented on the cluster. This leads to an approximation of the thermodynamic limit, in which the bulk problem is replaced by a finite-size cluster embedded in a mean-field host that is designed to represent the rest of the system. The basic assumption is that correlations are short ranged and contained within the cluster, so that the self-energy Σ(**k**, *iω*_*n*_) is well approximated by a cluster self-energy Σ(**K**, *iω*_*n*_), where **K** are the cluster momenta. One then calculates a coarse-grained Green's function


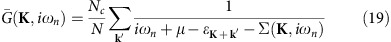


where *μ* is the chemical potential, which is tuned to give a fixed filling 〈*n*〉, and *N*_*c*_ is the number of sites in the cluster. For the 4 × 4 *N*_*c*_=16 site clusters that we choose, the sum averages over the momenta **k**′ in a square patch centred at 0 with sides of length *π*/2. This reduces the complexity of the problem to that of a finite cluster of size *N*_*c*_, which can be solved using QMC techniques. Here we use the CT-AUX quantum Monte Carlo algorithm developed by Gull *et al.*^10^ to calculate the self-energy 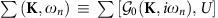 as a functional of the cluster-excluded propagator 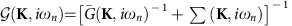 and the interaction *U*.

### Calculation of irreducible particle–particle vertex

In addition to the cluster single-particle Green's function *G*_*c*_(**K**, *iω*_*n*_), the QMC is also used to calculate the two-particle Green's function in the singlet particle–particle channel with zero centre of mass momentum and energy, *G*_*c*,2_(*K*, *K*′)≡*G*_*c*,2_(*K*, −*K*, *K*′, −*K*′), where *K*=(**K**, *iω*_*n*_) and 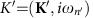. The irreducible particle–particle vertex 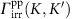 that enters in the DCA gap (equation (3)) is then extracted from the cluster Bethe–Salpeter equation


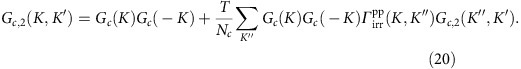


### Data availability

The data that support the findings of this study are available from the corresponding author upon request.

## Additional information

**How to cite this article:** Maier, T. A. *et al.* Pairing in a dry Fermi sea. *Nat. Commun.* 7:11875 doi: 10.1038/ncomms11875 (2016).

## Supplementary Material

Peer Review File
