# Supplementary material for: Pairing in a dry Fermi sea
Source: Nat Commun. 2016 Jun 17;7:11875. doi: 10.1038/ncomms11875 (PMC4915029; doi:10.1038/ncomms11875)
Supplement: Peer Review File [file ncomms11875-s1.pdf]

Editorial Note: this manuscript has been previously reviewed at another journal that is not operating a transparent peer review scheme. This document only contains reviewer comments and rebuttal letters for versions considered at Nature Communications.

Reviewers' comments:

Reviewer #1 (Remarks to the Author):

I have the read the author's response and think that they answered my queries sufficiently and also made efforts to satisfy Referee-II's comments. Hence I do not see any reason to cause further delay of this - in my opinion - very interesting and stimulating work.

Reply to the Reviewer #1:

We thank the referee for his time reviewing our manuscript and his recommendation for publication.
